# The Development of a Patient-Centered Digital Health Care Technology for Young Adults in Opioid Use Disorder Treatment: Qualitative Study

**DOI:** 10.2196/67401

**Published:** 2025-09-23

**Authors:** Karen Alexander, Madison Scialanca

**Affiliations:** 1 Friends Research Institute, 1040 Park Avenue, Suite 103, Philadelphia, PA, 21201, United States, 1 410 837 3977

**Keywords:** ecological momentary assessment, treatment barriers, opioid use disorder, opioid treatment programs, qualitative study, patient-centered, digital health care, digital care, digital technology, young adult, teenager, juvenile, proof-of-concept, self monitor, EMA, overdose, formative research, counselor, qualitative interview

## Abstract

**Background:**

Young adults, defined as individuals between the ages of 18 and 29 years, drop out of opioid use disorder (OUD) treatment more often than older adults. Premature treatment drop-out substantially increases fatal overdose risk. Self-monitoring through text messaging has been researched extensively among people with OUD to identify drop-out risk factors. Self-monitoring could potentially improve methadone treatment engagement among young adults, who are a population that is both hard to reach and more likely to use technology compared to older adults. Self-monitoring can increase risk factor awareness and help patients and counselors develop targeted coping strategies and treatment plans. However, embedding a discussion of risk factor information into existing counseling sessions has been limited and may offer a promising opportunity to improve engagement among young adults.

**Objective:**

This pilot proof-of-concept study examined the implementation of self-monitoring intervention, AWARE (Awareness and Response to the Environment), designed to bring attention to treatment drop-out risk factors among young adults and create discussion about risk factors with their existing treatment counselor.

**Methods:**

In this formative research, a convenience sample (N=8) of young adults (n=3, 38%) in methadone treatment, their counselors (n=3, 38%), and clinic leadership (n=2, 25%) were recruited from an opioid treatment program after referral from treatment staff. Participants were interviewed to obtain feedback as AWARE was developed. In semistructured interviews, perspectives regarding barriers to treatment for young adults and AWARE utility were obtained. Concurrently, 3 dyads of young adults (n=3, 38%) and counselors (n=3, 38%) piloted the intervention daily for 4 weeks.

**Results:**

The 3 consented young adults with OUD participants (n=2, 67% female; n=2, 67% Latino/a) were sent daily surveys for 28 days (53% overall completion rate). Young adults and counselors found AWARE relevant to their treatment experience and acceptable to complete over 4 weeks. The most reported daily stressors included concerns about the health and well-being of a family member, challenges with staying organized, and feeling overwhelmed by responsibilities without adequate support. In qualitative interviews, counselors and clinic leadership reported that AWARE presented a relevant, new way to engage young adults daily, in addition to weekly counseling sessions. Young adults felt that prompts sent by AWARE offered a type of social support they lacked, like “someone checking in on them.”

**Conclusions:**

Overall, young adult and counselor participants were able to engage in AWARE in a busy clinic environment, and participants and clinic leadership found it valuable. By addressing common stressors and providing a sense of social connection, AWARE may help fill a gap in support between counseling sessions. However, the study was limited by the small number of young adults engaging in methadone treatment. Further research is needed to refine the measures and methods of AWARE and evaluate its effectiveness.

## Introduction

### Background

Young adults, aged 18‐29 years, have the highest opioid use rate per capita [1]. Despite the efficacy of medications (eg, methadone and buprenorphine) to prevent morbidity and mortality related to opioid use, young adults with opioid use disorder (OUD) are less likely to initiate treatment [2,3] and more likely to drop out of treatment [4] compared to older age groups. A personalized behavioral treatment responsive to the individual needs of young adults may increase retention by addressing coping skills associated with young adults with OUD stressors and thoughts of leaving treatment [5-8].

### Addressing Risk Factors Through Ecological Momentary Assessment

The scientific understanding of risk assessment in a real-world context has been advanced by ecological momentary assessment (EMA) research methods [9,10]. EMA is a data collection technique requiring frequent self-monitoring of symptoms, thoughts, and social interactions, enabling the identification of unfolding patterns of mental states and behavior [11]. Self-monitoring through text messaging (an EMA method) has been used extensively among people with OUD to identify treatment drop-out risk factors through pattern recognition [12-17].

People with OUD have differing retention trajectories based on levels of stress [18,19]. The variability of stress, not the averages at points in time, best predicts craving and future opioid use [20]. According to self-management and cognitive relapse prevention theories, greater attention to and awareness of mental states may lead to improvements [21,22]. Drop-out risk factors like stress may normally be unconscious to a person, but self-monitoring can bring them to the forefront and, thus, increase the opportunity for the patient and counselor to develop more specific plans, coping strategies, and more control [23]. It is likely that automatic responses to stress, when brought to cognitive awareness, become easier to address and extinguish [24].

Previous research has incorporated EMA data within OUD treatment to engage participants through text messages to remind participants of appointments [25], mobile health (mHealth) applications to deliver therapy [26-28], and web-based software to access and deliver cognitive behavioral therapy (CBT) [29]. However, few digital interventions are described in the literature that incorporate EMA data into counseling sessions within an existing opioid treatment program (OTP). Integrating real-time patient-generated data at the point of care has the potential for a significant impact by informing risk assessment and augmenting existing treatment.

### Study Purpose

The present study developed and pilot-tested an intervention tailored for young adults with OUD called Awareness and Response to the Environment (AWARE). AWARE used Research Electronic Data Capture (REDCap; Vanderbilt University) software to send EMA surveys on a study-provided phone, collecting patient-generated data on antecedents of treatment drop-out that could be integrated into weekly counseling sessions. The purpose of this paper is to (1) describe the use of AWARE by young adults with OUD and treatment staff, (2) detail the perceived barriers and facilitators of treatment engagement, and (3) describe how AWARE may address identified barriers. This pilot, proof-of-concept project will lay the foundation for a future clinical trial of AWARE.

### Theoretical Framework

**Figure 1.**
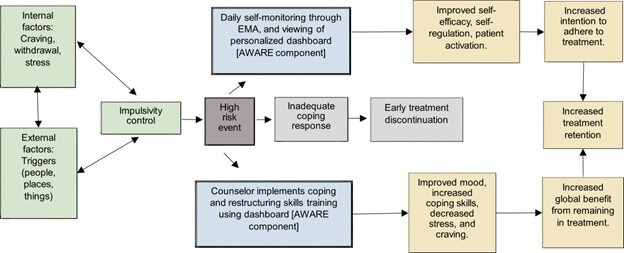
Integration of AWARE components with theoretical frameworks. AWARE: Awareness and Response to the Environment; EMA: ecological momentary assessment.

The conceptual framework for AWARE draws from self-management and cognitive-behavioral relapse prevention theories, which are applied to the retention stage of the OUD Cascade of Care [30]. To target improvements in the retention stage, self-efficacy beliefs (“I’m going to the clinic today”) and adherence (“I received medication today”) to medication are crucial [31,32]. Interventions underpinned by self-management theory in HIV and substance use research have shown success in increasing medication receipt and adherence [33,34]. Self-management theory emphasizes that improvements in self-efficacy beliefs can result in better decision-making and intention to adhere to healthy behaviors [33-35]. Cognitive-behavioral relapse prevention theory demonstrates that relapse (defined as a setback in progressing toward recovery) is not a binary event but, rather, a dynamic, fluctuating process (see Figure 1) [7,26]. The rationale for integrating these 2 theories stems from the utility of self-management–based interventions in chronic disease and the chronic, relapsing nature of addiction [34,36]. Improved self-management skills for someone with OUD may prevent future relapses but also could improve daily cravings, negative mood, and stress, which may reflect a greater benefit to an individual than abstinence from drugs alone [37]. These personal benefits may also positively reinforce treatment adherence, thereby making the work of recovery more appealing to participants [38].

## Methods

### Setting and Design

Young adults with OUD and treatment staff recruitment took place at an urban, Northeastern United States OTP that offers methadone or buprenorphine and individual and group counseling informed by CBT, using the National Institute on Drug Abuse (NIDA) CBT manual [39]. Each incoming patient receives an individual counselor with a caseload of 25‐30 patients. The OTP treated 426 patients in 2023 for OUD with either methadone (93%) or buprenorphine (7%). Approximately 10% of the patient population were young adults in 2023. The program does not currently use patient-centered digital health care technology. Medications administered and dispensed, as well as doses and drug screening results, are recorded in an electronic health record (EHR). Urine samples are obtained randomly at least twice per month, and patients are not discharged for drug use alone. Rather, continued opioid or other drug use may impact clinical decisions regarding take-home medication, dosage, and counseling approaches. Reasons for discharge are noted in the EHR. During our pilot study, we capitalized on already existing resources, including existing counselors, to adapt AWARE into the opioid treatment center structure. This study used a qualitative approach, which is appropriate for formative work. This article adheres to the COREQ (Consolidated Criteria for Reporting Qualitative Research) regarding key data collection and analysis aspects.

### AWARE Intervention

AWARE was developed as a self-monitoring, mobile phone–based intervention that couples EMA surveys with counselor feedback during weekly CBT sessions. The goal of AWARE is to augment standard care, which includes medication, through identifying and responding to high-risk events or situations known to predict drop-out. The EMA structure, content, and timing in this project are informed by longitudinal studies that sought to predict early treatment dropout using EMA [18,40-46]. For this pilot study, the Daily Hassles Survey [18], a list of external stressors, was used to identify stressors particular to young adults with OUD. Weekly data summaries were provided to the participant and their counselor via email (see Table 1).

**Table 1.** Awareness and Response to the Environment intervention integration of EMA data into counseling sessions for young adults with opioid use disorder.

Participation and roles of participant and counselor	Activities	Theoretical construct
Participant (daily) self-monitors via EMA, views dashboard, real-world practice of skills	Identifies and examines thoughts, symptoms, triggers of drop-out; practices coping skills; practices problem solving	Self-efficacy, self-regulation, patient activation; coping, relapse or drop-out prevention; self-efficacy, relapse prevention;
Study counselor (weekly) views dashboard, analyzes patterns	Identifies and examines thoughts, symptoms, triggers of drop-out; identifies contextual issues affecting intention to stay in treatment; teaches and leads rehearsal of coping skills (emotional and trigger coping); engages in problem solving	Self-efficacy, self-regulation; self-efficacy, self-regulation; coping, relapse or drop-out prevention; coping, relapse, or drop-out prevention

### Eligibility Criteria

The study recruited young adults with OUD who were (1) aged 18‐29 years, (2) receiving counseling during OUD treatment, and (3) English speaking. We excluded young adults with OUD if they exhibited (1) active suicidal ideation and/or (2) active psychosis. Inclusion criteria for treatment provider/staff interviews included (1) counselors with a client enrolled in AWARE or (2) clinic leadership.

### Recruitment and Screening Procedures

The research assistant approached eligible young adults with OUD participants during dosing, asking them if they would like to participate in a study regarding a mobile phone–based intervention. The research assistant then obtained written informed consent and conducted the baseline interview. Participants were then oriented to the EMA survey procedures. Treatment center staff were recruited at a kick-off breakfast event at the treatment center. Staff provided verbal consent to the interviews before their interview began. Of the 14 young adult clients enrolled in the opioid treatment program, 8 were contacted and the research team never received a response; 3 were discharged from the treatment center before contact could be made, and 3 were successfully enrolled.

### Measures

All study participants completed a brief demographic questionnaire at baseline. In addition, young adults with OUD participants completed the daily stressors questionnaire. Qualitative interviews were performed using a semistructured guide before and after AWARE implementation. All participants were asked key questions regarding perceived barriers to treatment for young adults with OUD and the utility of AWARE in improving treatment engagement. Suggestions for improvement, as well as content or delivery methods, were obtained. Participants received a US$50 gift card upon completion of the qualitative interview. Young adults with OUD participants were paid US$50 in cash at the beginning and end of the AWARE intervention, and they also received a study phone if desired.

### Data Analysis

Interview data were analyzed using thematic analysis methods: generating initial codes, searching, reviewing, defining, and naming subthemes, and identifying basic and global themes. The research team consisted of a master’s level Research Assistant and a PhD-prepared nurse scientist, each with extensive research and clinical experience working with young adults with OUD. Transcripts were reviewed independently by a master’s prepared research assistant and a PhD-prepared investigator using rigorous and accelerated qualitative data reduction (RADaR) and content analysis [47]. A series of spreadsheets was created to produce short, concise data tables. From the discussion of these tables, a consensus was reached on the content and relevance of themes identified. The identified daily stressors were tabulated using frequency of identification and ranked from most identified to least identified.

### Ethical Considerations

The study was approved by both WCG Institutional Review Board and the City of Philadelphia Department of Public Health Institutional Review Board (IRB# 2024-07). We obtained written, informed consent from all patient participants and verbal consent from the counselor and clinic leadership to be interviewed. All study data were deidentified before analysis. Participants were assigned unique study IDs, and no identifying information (eg, names, addresses, and phone numbers) was stored with survey or interview responses. Data collected through electronic platforms (eg, REDCap) were stored on secure, password-protected cloud-based storage systems compliant with institutional and Health Insurance Portability and Accountability Act (HIPAA) standards. Access to identifiable data was restricted to key research personnel and monitored through audit logs. All qualitative interview recordings were transcribed with identifying details removed and securely deleted after transcription. Findings are reported in aggregate form to ensure individual confidentiality. Patient and staff participants were paid US$50 for completing baseline and follow-up interviews at the beginning and end of the 4-week intervention period.

## Results

### Overview

The 3 consented young adult participants with OUD (n=2, 67% female; n=2, 67% Latino/a) were sent daily surveys for 28 days (53% overall completion rate), and only 2 young adult participants with OUD finished the study. All 3 counselors received a weekly data summary. Young adults with OUD and counselor ratings of the usability of the platform were favorable; all agreed with statements that the daily surveys were clinically useful, easy to navigate, and relevant to the treatment experience. The young adults with OUD and treatment center staff interviews centered on the previously identified themes of treatment engagement stressors and barriers and the feasibility and usability of AWARE to promote treatment engagement.

### Participant Characteristics

Three young adult participants with OUD consented to participate in the study. The first participant was a Latina woman, aged 27 years, living in her own home with her partner and child. The second participant was a Latino man living in a family member’s house. The third participant was a Black woman experiencing ongoing homelessness; however, she was lost to follow-up after responding to one week of surveys. All participants received a study phone to complete the AWARE surveys.

Five treatment center staff were recruited. All but one of the counselors had less than 6 years of experience as a drug and alcohol counselor. One clinic administrator had over 20 years of experience working in therapy within opioid treatment programs. Two treatment center staff identified as Black, one identified as Asian, and 2 identified as White. Treatment center staff interviewed primarily identified as women, except for one man.

### Treatment Engagement Stressors and Barriers for Young Adults

Over the 28-day period, young adults with OUD most frequently reported stressors related to family-related obligations (10 responses), having to do things without help (9 responses), and being organized (9 responses). Other common stressors included the health of a family member, physical care, and medical abilities (each 8 responses). In contrast, children, partners, and boredom (“having enough to do”) were reported least often (0–3 responses). Overall, stressors spanned family, financial, health, and daily-life domains, with practical and relational demands emerging as the most prominent. In qualitative interviews, 2 young adults with OUD participants identified “not having much to do” and not having any social connections outside of the clinic as a frequent stressor. The most frequently reported daily stressors were identified as “the health and well-being of a family member,” “being organized,” and “having too many things to do without help.”

One participant described the care she provides daily for her child and her partner, who works night shift and is also a patient at the methadone clinic.

*I have to be up and ready by 7:30 AM. [My partner is] outside and then I know we’re coming to the clinic and he’s tired, so he has to go home and sleep till he goes to work. So it’s like I know I can’t do nothing Monday through Friday but cater to him and the baby.*

[Participant 1]

Each young adult with OUD reported in their interviews that they had difficulty in the past with clinic regulations and the clinic environment. This was not the first treatment episode for any young adults with OUD participants. The social environment around the clinic was not seen as positive. Most participants reported going to and from the clinic and not spending time with peers.

*Well, it’s like with this time, this isn’t my first rodeo, so I know what to do this time. So I know this might not be the best answer, but this works for me. I isolate and it’s not good. I isolate because I don’t live in a nice neighborhood. There’s drugs everywhere, but I don’t like, it’s definitely my mindset. I just go straight home and I try to keep a one track mind, but I mean, that wasn’t good for me to do it back then.*

[Participant 3]

Young adults with OUD participants also reported that they did not have much to do and making money at a job posed a problem, as they feared they might use the money for drugs.

*Because you got to understand the last 10 years of my life getting high was my hobby, my interest. So when I just stop, you do feel lost, then that’s when depression and being miserable, that all kicks in. You just don’t know what to do. But I just got to find new shit to do, to keep me occupied, and I don’t even know what to do right now. You know what I mean? It’s weird. I feel like I’m learning myself. I feel like how I felt when I was in high school. I am learning, trying to find myself, [trying to find things] that interest me instead of getting high.*

[Participant 3]

Treatment center staff had a similar perspective to young adults with OUD regarding stressors and barriers to treatment engagement. All 5 treatment staff participants mentioned housing as a distinct barrier to stay in treatment. However, treatment center staff also highlighted issues related to childhood trauma, motivation, and family disconnection as barriers to maintaining recovery.

*A lot of [clients] are not close with their families anymore, so I mean they don’t have anything to look forward to. Holidays, everything is just a regular day to them, so because they don’t have the motivation or the push, it makes it very difficult to keep them engaged on sobriety. They don’t see a reason to be sober.*

[Staff 2]

Difficulties staying away from drug use were linked by several treatment center staff to the environment in which participants lived and socialized. According to treatment center staff, there was little motivation to stay away from drugs and staying in treatment was lacking for many participants, due to a lack of positive relationships. Treatment staff also mentioned that the relationships that they do have at the treatment center are not always positive and that many times staying away from people is a strategy young adults employ in order to stay engaged in treatment.

### AWARE Feasibility, Acceptability, and Usability

Young adults and treatment center staff found AWARE to be relevant, acceptable, and feasible to complete over 4 weeks, although completion rates varied. Of the 3 young adults with OUD participants who began the AWARE intervention, only 2 completed over 90% of the 28 surveys sent. The third participant completed less than half of the surveys sent. Participants reported that it took 5‐10 minutes to complete their daily survey and that AWARE was “easy to use” and “didn’t take much time.”

*I think it pretty much everything made sense the way [AWARE] was set up because there wasn’t too little questions or too much. It was kind of perfect to just reflect or more or less.*

[Participant 1]

Young adults with OUD participants felt that AWARE was offering a type of social support, like “someone checking in on them.” One participant mentioned they were able to keep in contact with their parole officer as a result of having a study phone.

Clinic leadership and counselors reported that they felt AWARE presented a relevant, new way to engage young adults on a daily basis, in addition to weekly meetings. They also mentioned that AWARE allowed for anonymous reflection on the part of their clients. Counselors also appreciated that AWARE enabled weekly discussion of stressors based on patient-generated, real-time data.

*[You] sent me a summary of my client’s information, and I thought that was really helpful. I can actually bring this up in session and see what exactly going on. Because usually in session, it’s kind of hard to pull that out of him or it’s the generic, “how are you doing,” “how’s your week been,” and “it’s fine, fine.*”

[Staff 4]

Treatment center staff also identified AWARE as an intervention that provides check-ins with their clients, addressing the scarcity of relationships in their clients’ lives. AWARE extended the reach of a positive relationship at the treatment center through automated messaging and required response.

*And I think it’s great because it’s daily, because in a way it gives them something to feel a part of. Like somebody cares, there’s a regular check-in. A lot of them don’t have anything, don’t have anybody asking them anything. Don’t have any conversations, nobody checking in.*

[Staff 2]

## Discussion

### Principal Findings

This is the first study to develop a patient-centered digital health care technology specifically for young adults in OUD treatment to promote retention through client-counselor engagement with EMA data. This pilot study sought to explore the use of AWARE among young adults with OUD and counselors to address the barriers and facilitators to treatment engagement. We aimed to identify barriers to treatment engagement and assess the potential of AWARE to address these barriers through real-time, technology-based support. Although preliminary, our findings provide valuable insights that lay the groundwork for a future clinical trial designed to rigorously evaluate the effectiveness of AWARE in improving treatment engagement and outcomes for this hard-to-reach population.

Initial findings from this pilot project indicate that delivery of daily surveys to young adult participants and the delivery of weekly summaries to counselors regarding stressors is not only possible but relevant to all involved. Previous research has found that people with OUD want a more personalized approach to treatment [48]. Methadone treatment delivery is largely unchanged since initial federal regulations and policies were established in 1972 [49]. New delivery methods are needed to promote treatment retention among young adults who demonstrate the highest opioid use rate per capita and the highest treatment drop-out rates [50-52]. To engage in treatment, young adults and others need treatment to be personalized and relevant to their circumstances (grounded in real-world experiences) [48]. Previous mHealth research for people with OUD has not specifically targeted young adults who are hard to reach and underrepresented in treatment [53-55]. Future research should focus on engaging out-of-treatment populations of young adults with new delivery innovations.

Young adult participants with OUD identified several stressors that may interfere with treatment engagement, including housing, transportation, drug use, and family obligations. Improved self-management in response to stress, craving, and negative mood is particularly crucial for young adults who are in a stage of exploration and identity formation and who often experience high levels of impulsivity in response to external and internal triggers [56,57]. Two young adults with OUD participants identified “not having much to do” and not having any social connections outside of the clinic as frequent stressors. Among individuals with OUD, loneliness has been recognized as a factor that drives substance use and increases cravings, and it is linked to significant triggers that can lead to relapse [58]. AWARE was able to reach out to participants on a daily basis and make them feel supported and less alone. It is possible that more contacts with positive relationships, through an intervention like AWARE, could not only increase treatment engagement but also begin to address social isolation.

Interventions that integrate a response to patient-generated data within existing substance use disorder treatment are more effective than self-monitoring alone at improving treatment retention [59].  However, patient-generated data has not been integrated within a direct point-of-care system workflow to inform treatment response to address drop-out risk factors at an opioid treatment center. AWARE presents an opportunity to integrate real-time patient-generated data at the point of care, which has the potential for significant impact by informing risk assessment and increasing opportunities for patient engagement and shared decision-making regarding treatment options. Such an approach promises to increase early treatment retention and thereby improve the quality of care and reduce the risk of overdose death.

### Limitations

All participants received the intervention. Future studies will be trials of AWARE and will include control and intervention groups to test the effect of AWARE. The small sample size of this specific study tested the recruitment strategy and highlighted the small number of young adults in treatment for OUD at a large, urban opioid treatment center at one point in time. As a result, findings are likely influenced by selection bias, particularly toward young adults who were more motivated to engage in research. The results may not reflect the broader population of young adults in OUD treatment. In addition, due to the limited sample size, thematic saturation was not achieved in qualitative interviews; findings should be interpreted as preliminary and exploratory, intended to inform future refinement and testing of the intervention.

### Conclusion

Overall, young adults with OUD and treatment staff participants were able to engage in AWARE in a busy OTP environment, and participants found it valuable. By addressing common stressors and providing a sense of social connection, AWARE may help fill a gap in support between counseling sessions for a hard-to-reach and underrepresented population in methadone treatment. Further research is needed to refine the measures and methods of AWARE and evaluate its effectiveness.

## Abbreviations

AWARE: Awareness and Response to the Environment
CBT: cognitive behavioral therapy
COREQ: Consolidated Criteria for Reporting Qualitative Research
EHR: electronic health record
EMA: ecological momentary assessment
HIPAA: Health Insurance Portability and Accountability Act
mHealth: mobile health
NIDA: National Institute on Drug Abuse
OTP: opioid treatment program
OUD: opioid use disorder
RADaR: rigorous and accelerated data reduction
REDCap: Research Electronic Data Capture
